# Molecular circadian clock disruption in the leukocytes of individuals with type 2 diabetes and overweight, and its relationship with leukocyte–endothelial interactions

**DOI:** 10.1007/s00125-024-06219-z

**Published:** 2024-07-10

**Authors:** Clara Luna-Marco, Deédeni Devos, Julia Cacace, Meylin Fernandez-Reyes, Pedro Díaz-Pozo, Juan D. Salazar, Eva Solá, Carlos Morillas, Milagros Rocha, Víctor M. Víctor, Susana Rovira-Llopis

**Affiliations:** 1 Service of Endocrinology and Nutrition, University Hospital Doctor Peset, Foundation for the Promotion of Health and Biomedical Research in the Valencian Region (FISABIO), Valencia, Spain; 2 Department of Physiology, University of Valencia, INCLIVA (Biomedical Research Institute Valencia), Valencia, Spain; 3 CIBERehd – Department of Pharmacology, University of Valencia, Valencia, Spain

**Keywords:** Cardiovascular disease, Circadian rhythm, CLOCK, Leukocyte–endothelial interactions, Type 2 diabetes

## Abstract

**Aims/hypothesis:**

Alterations in circadian rhythms increase the likelihood of developing type 2 diabetes and CVD. Circadian rhythms are controlled by several core clock genes, which are expressed in nearly every cell, including immune cells. Immune cells are key players in the pathophysiology of type 2 diabetes, and participate in the atherosclerotic process that underlies cardiovascular risk in these patients. The role of the core clock in the leukocytes of people with type 2 diabetes and the inflammatory process associated with it are unknown. We aimed to evaluate whether the molecular clock system is impaired in the leukocytes of type 2 diabetes patients and to explore the mechanism by which this alteration leads to an increased cardiovascular risk in this population.

**Methods:**

This is an observational cross-sectional study performed in 25 participants with type 2 diabetes and 28 healthy control participants. Clinical and biochemical parameters were obtained. Peripheral blood leukocytes were isolated using magnetic bead technology. RNA and protein lysates were obtained to assess clock-related gene transcript and protein levels using real-time PCR and western blot, respectively. Luminex XMAP technology was used to assess levels of inflammatory markers. Leukocyte–endothelial interaction assays were performed by perfusing participants’ leukocytes or THP-1 cells (with/without CLK8) over a HUVEC monolayer in a parallel flow chamber using a dynamic adhesion system.

**Results:**

Participants with type 2 diabetes showed increased *BMAL1* and *NR1D1* mRNA levels and decreased protein levels of circadian locomotor output cycles kaput (CLOCK), cryptochrome 1 (CRY1), phosphorylated basic helix-loop-helix ARNT like 1 (p-BMAL1) and period circadian protein homologue 2 (PER2). Correlation studies revealed that these alterations in clock proteins were negatively associated with glucose, HbA_1c_, insulin and HOMA-IR levels and leukocyte cell counts. The leukocyte rolling velocity was reduced and rolling flux and adhesion were enhanced in individuals with type 2 diabetes compared with healthy participants. Interestingly, inhibition of CLOCK/BMAL1 activity in leukocytes using the CLOCK inhibitor CLK8 mimicked the effects of type 2 diabetes on leukocyte–endothelial interactions.

**Conclusions/interpretation:**

Our study demonstrates alterations in the molecular clock system in leukocytes of individuals with type 2 diabetes, manifested in increased mRNA levels and decreased protein levels of the core clock machinery. These alterations correlated with the impaired metabolic and proinflammatory profile of the participants with type 2 diabetes. Our findings support a causal role for decreased CLOCK/BMAL1 activity in the increased level of leukocyte–endothelial interactions. Overall, our data suggest that alterations in core clock proteins accelerate the inflammatory process, which may ultimately precipitate the onset of CVD in patients with type 2 diabetes.

**Graphical Abstract:**

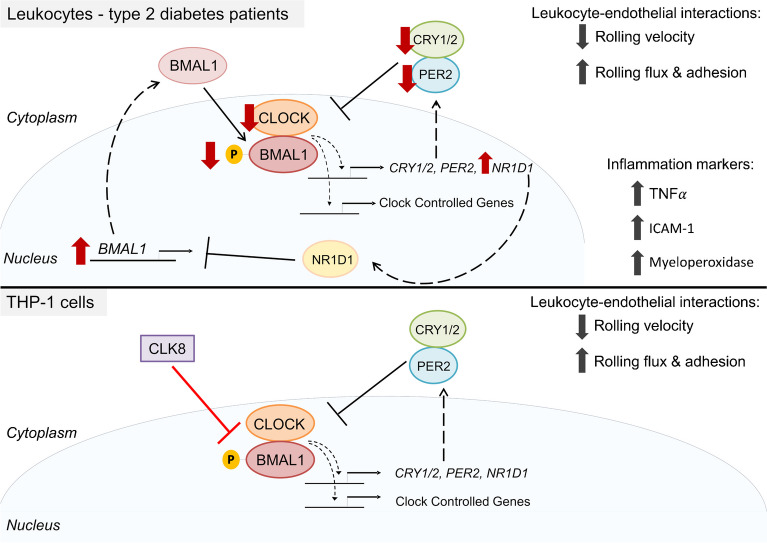

**Supplementary Information:**

The online version contains peer-reviewed but unedited supplementary material available at 10.1007/s00125-024-06219-z.


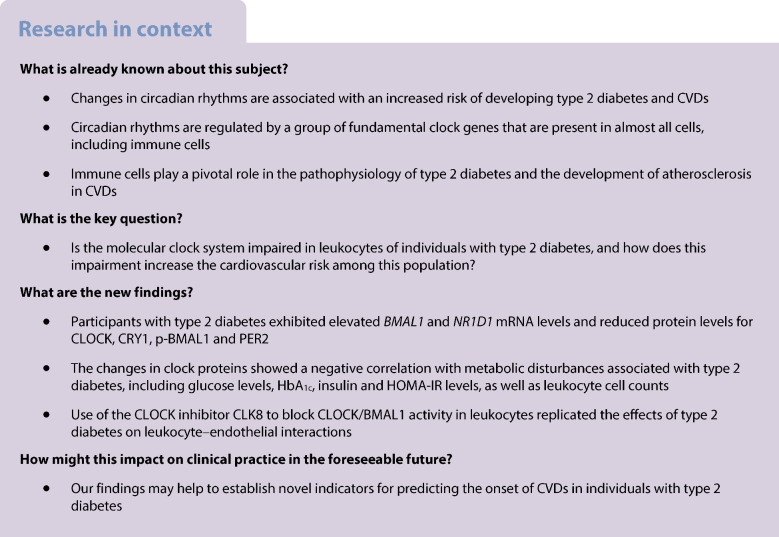


## Introduction

The prevalence of type 2 diabetes is increasing globally and is reducing life expectancy, making it a significant health concern. In 2021, it was projected that 537 million adults were living with diabetes, accounting for approximately 10.5% of the world’s population [1].

CVD remains the major determinant of premature mortality in patients with type 2 diabetes [2]. Type 2 diabetes presents systemic inflammation that arises as a result of chronic hyperglycaemia [3]. In addition, diabetes-associated dyslipidaemia and oxidative stress promote development of atherosclerosis, as LDL-cholesterol particles are targets of oxidation and are a key factor in the process. Under these conditions, cytotoxic damage occurs to endothelial cells, promoting the release of proinflammatory cytokines and the recruitment of innate and adaptive immune cell populations into atherosclerotic lesions. Finally, migration of monocytes into the sub-endothelial space and uptake of oxidised LDL leads to foam cell formation [4].

An unhealthy diet and sedentary lifestyle are major risk factors for the development of type 2 diabetes and CVD [5]. In recent years, alterations in daily rhythms, such as those suffered by shift workers, have been shown to be related to a greater risk of developing obesity, type 2 diabetes and the metabolic syndrome [6–8]. Recent evidence also suggests that circadian alignment of food intake provides metabolic benefit in terms of reduced glycaemic parameters [9]. The mammalian circadian rhythm is controlled by a central clock in the hypothalamus and a multitude of peripheral clocks in other areas of the brain and peripheral tissues [10, 11]. The molecular basis of the mammalian circadian clock consists of a transcriptional/translational autoregulatory feedback loop that involves several clock genes, including *CLOCK*, *BMAL1*, *PER1*/*2*/*3* and *CRY1*/*2*, among others. Circadian rhythms are essential for the maintenance of physiological functions such as glucose metabolism, and their alteration has been shown to be associated with the development of insulin resistance [12], although the precise mechanism involved is unknown.

There is circadian rhythmicity in gene expression profiles in human peripheral blood mononuclear cells (PBMCs), and the transcript levels of core clock genes in PBMCs of healthy participants follow a 24h cyclical pattern [13, 14]. Indeed, clock gene expression profiles in human PBMCs have been proposed as a tool for assessing circadian rhythm in humans [15]. Circulating PBMCs are sensors of metabolic stress and bioenergetic markers [16]. These cells play a key role in the onset of diabetes, as they are the precursors of the adipose-resident immune cells that promote inflammation during obesity, which eventually leads to type 2 diabetes [17]. Moreover, their interaction with and adherence to the endothelium is an indicator of subclinical atherosclerosis and precedes the onset of CVD [18]. Leukocyte–endothelial interactions are enhanced in type 2 diabetes patients [19, 20], but the underlying molecular pathways that trigger these interactions are only partially understood [20–22].

On this basis, we aimed to study whether there are alterations in the expression of circadian rhythm genes in the PBMCs of patients with type 2 diabetes, and to explore the mechanism by which these genes participate in the pathophysiology of type 2 diabetes. We specifically evaluated whether this disruption affects the interaction between leukocytes and endothelial cells that, together with chronic low-grade inflammation, underlies the pathophysiology of type 2 diabetes.

## Methods

### Participant recruitment and measurement of clinical parameters

This is an observational cross-sectional study. Participants with type 2 diabetes (*n*=25) and healthy control volunteers (e.g. health workers’ family members, friends and acquaintances; *n=*28) were recruited at the Endocrinology and Nutrition Service of the Hospital Universitario Doctor Peset (Valencia, Spain). Self-reported sex and ethnicity data were collected. Eligibility criteria for participants were as follows: adults up to 70 years of age; for participants in the type 2 diabetes group, diagnosis of type 2 diabetes was based on the ADA guidelines [23] and known time of evolution of the disease greater than 5 years was required, which is representative of the disease population in our setting. The exclusion criteria included the presence of other diseases, such as morbid obesity, or any autoimmune, malignant, organic, haematological, inflammatory or infectious disease. The protocol was approved by the hospital’s clinical research ethics committee (ID: 131.22), and was performed according to the ethical principles of the Declaration of Helsinki. Participants provided written, informed consent and biochemical determinations of blood samples were performed by the hospital’s clinical analysis service.

### Leukocyte extraction and isolation

A blood sample was extracted from participants under fasting conditions between 08:00 and 09:00 hours using EDTA-coated tubes. PBMCs were isolated using MACSprep kits (130-115-169; Miltenyi Biotec, Germany) according to the manufacturer’s instructions, and cell density was measured using a LUNA-FL cell counter (Logos Biosystems, Korea). Cells were immediately used for in vivo experiments (leukocyte–endothelium assays) or frozen at −80°C until use for RNA and protein extraction. Due to the limited amount of blood and the number of leukocytes extracted, it was not always possible to perform all experiments for every participant. Participants’ leukocytes were randomly assigned to at least one of the techniques involving leukocyte samples. Masking was carried out for all the experiments involving leukocyte samples, by identifying samples with a number.

### Cell lines and cell culture

THP-1 (TIB-202; ATCC, USA), a human monocyte cell line isolated from peripheral blood, was used for in vitro studies with the circadian rhythm inhibitor CLK8 (3224; Axon Medchem, the Netherlands). Cells were cultured in RPMI medium (pH 6.8–7.2: L0500–509; Biowest, France) supplemented with 10% FBS (S1400; Biowest), 1% penicillin/streptomycin and 1% fungizone for expansion. The cell line was regularly verified to be free of mycoplasma. They were incubated at 3×10^5^cells/ml with 20 µmol/l CLK8 for 48h. A vehicle control with DMSO was also included in the protocol. After this incubation period, cells were collected by centrifugation (200 *g*, 5 min, 23°C) and resuspended in RPMI medium supplemented with 10% vol./vol. FBS, 1% penicillin/streptomycin, 1% glutamine and 1% sodium pyruvate at a density of 10^6^cells/ml. Interactions of the THP-1 cells with an endothelial monolayer were studied as described below.

### Evaluation of adhesion and inflammatory molecules

Levels of TNF-α, intercellular adhesion molecule 1 (ICAM-1) and myeloperoxidase (MPO) were measured in serum obtained by centrifuging blood collected in gel serum separator tubes (1500 *g*, 10 min, 4°C). A Luminex 200 analyser (Luminex, USA) was used to determine the serum levels according to the procedures provided by the MILLIPLEX kit manufacturer (Millipore, USA). Samples below the detection threshold were excluded from the analysis.

### RNA extraction and reverse transcription quantitative PCR (RT-qPCR)

RNA was extracted from PBMCs (2.5×10^6^ cells) preserved at −80°C in RNA*later* stabilisation solution (AM7020; Thermo Fisher, USA) and previously obtained from participants with and without type 2 diabetes using Ribospin RNA extraction kits (304-150; GeneAll, Korea). A Nanodrop 2000 spectrophotometer (Thermo Fisher) was used to evaluate RNA quantity and quality (*A*_260_/*A*_280_≈2). RNA samples (1000 ng) were converted to cDNA using RevertAid first-strand cDNA synthesis kits (K1622; Thermo Fisher) in three steps (5 min at 25°C, 60 min at 42°C, 5 min at 70°C) (*V*=20 µl). The genes in which we were interested (*CLOCK*, *CRY1*, *BMAL1*, *CRY2*, *NR1D1* and *PER2*) were further amplified and quantified using a 7500 fast real-time PCR system thermocycler (Thermo Fisher) and FastStart Universal SYBR Green (491385001; Sigma-Aldrich, USA) (10 min at 95°C, 40 cycles of 10 s at 95°C and 30 s at 60°C, melting curve: 15 s at 95°C and then temperature is incrementally increased 1°C per cycle starting at 60°C and reaching 95°C in approximately 30 min; volume=10 µl). Samples were analysed in duplicate. The primer sequences were designed using NCBI BlastN and are shown in electronic supplementary material (ESM) Table 1. Results were normalised to the expression of the housekeeping gene 18S RNA, and are expressed as ΔΔ*C*_t_ values. RNAseAway (Thermo Fisher) was used during all procedures to avoid DNase and RNase activity, and ‘no template’ controls were added during the qPCR process.

### Western blot

Protein detection was performed by western blot. Due to the limited amount of participants’ leukocytes available, leukocytes from at least six participants per group were included in western blots. The characteristics of these subgroups of participants are shown in ESM Table 2. Isolated PBMCs were mechanically and chemically lysed, and protein was extracted using Pierce RIPA buffer (89900; Thermo Fisher) combined with a protease inhibitor cocktail (1861284; Thermo Fisher). After protein quantification using a Pierce BCA protein kit (23255; Thermo Fisher), 25µg of protein was separated on a Novex WedgeWell 8% Tris-glycine gel (XP00085BOX; Thermo Fisher) at 150 V for 1h. The proteins were transferred to a nitrocellulose membrane (1620167; Bio-Rad, USA) at 400 mA for 1h. An immunochemiluminescent approach was used to detect the proteins of interest. Primary antibodies against circadian locomotor output cycles kaput (CLOCK; 1:500 dilution, rabbit polyclonal, Abcam cat. no. ab3517, RRID: AB_303866), cryptochrome 1 (CRY1; 1:500 dilution mouse monoclonal, Abcam cat. no. ab54649, RRID: AB_2276575), phosphorylated basic helix-loop-helix ARNT like 1 (p-BMAL1; 1:1000 dilution, rabbit, Cell Signaling Technology cat. no. 13936, RRID: AB_2798348), BMAL1 (1:2000 dilution, rabbit polyclonal, Abcam cat. no. ab3350, RRID: AB_303729), nuclear receptor subfamily 1 group D member 1 (NR1D1; 1:2000 dilution rabbit polyclonal, Proteintech cat. no. 14506-1-AP, RRID:AB_11182604), CRY2 (1:2000 dilution rabbit polyclonal, Abcam cat. no. ab93802, RRID: AB_2083986), period circadian protein homologue 2 (PER2; 1:500 dilution, rabbit monoclonal, Abcam cat. no. ab179813) and actin (1:2000 dilution, rabbit polyclonal, Sigma-Aldrich cat. no. A5060, RRID: AB_476738) were used together with goat anti-mouse IgG (1:200 dilution, Thermo Fisher Scientific cat. no. 31430, RRID: AB_228307) or goat anti-rabbit IgG (1:2000 dilution, Vector Laboratories cat. no. PI-1000-1, RRID: AB_2916034) horseradish peroxidase-conjugated secondary antibodies. All antibodies, primary and secondary, were diluted in 5% milk in TBS with Tween 20 (TBST), except p-BMAL which was diluted in 5% BSA in TBST. All antibodies had been validated previously as stated in their datasheet. The chemiluminescent signal was developed using either SuperSignal West Pico or SuperSignal West Femto (34580 and 34095; Thermo Fisher). Images were obtained using a Fusion FX system (Vilber Lourmat, France) and processed using Bio1D software (Vilber Lourmat, France). All results were normalised to actin protein expression, except that of p-BMAL1, which was normalised to BMAL1 expression.

Due to the limited number of leukocytes in the blood samples, lysates from each patient’s leukocyte pellet achieved very limited protein concentration. Therefore, we tried to maximise the number of proteins detected for each sample by blotting each gel with two different antibodies sequentially. The two targets were always clearly differentiated by size, and stripping of the membrane was performed between the two incubations to avoid cross-reaction. For this reason, the same control was used to normalise the protein signal in some cases; this is indicated in the figure legend.

### Leukocyte–endothelium interaction assays

HUVEC/TERT2 cells (CRL-4053; ATCC) were cultured until confluence in 35 mm ×10 mm culture dishes (430165; Corning) with RPMI medium (pH 6.8–7.2) supplemented with 10% vol./vol. FBS. Additionally, HUVECs were treated (or not) with 1.25 ng/ml TNF-α for 4 h before the experiment was initiated. Interactions between participants’ leukocytes or THP-1 cells and endothelial cells were studied in a parallel flow chamber by means of a dynamic adhesion system. Following leukocyte isolation or THP-1 collection, 10^6^cells/ml were resuspended in adhesion medium containing 10% vol./vol. FBS, 1% penicillin/streptomycin, 1% glutamine and 1% sodium pyruvate. Cells were then perfused over the endothelial monolayer at 0.3 ml/min for 5 min inside the flux chamber, and recorded using a Nikon Eclipse Ts2R microscope (Nikon, Japan). Rolling velocity, rolling flux and adhesion parameters were calculated using Tracker (free software from physlets.org/tracker/; access date: 23 October 2022).

### Statistical analysis

Statistical analysis was performed using SPSS version 27.0 (SPSS Statistics, USA) and GraphPad (www.graphpad.com). *p* values <0.05 were considered statistically significant. Normally distributed data were compared using Student’s *t* test, and the Mann–Whitney *U* test was applied to non-normally distributed data. Two-way ANOVA was used to compare the mean differences in the presence of two factors. All tests were unpaired except those applied to leukocyte–endothelium interaction assays using THP-1 cells. The χ^2^ test was used to compare proportions, and the influence of age and BMI was tested and corrected using a general linear model. Spearman's *r* was used for correlation studies comparing clinical parameters and protein expression levels.

## Results

### Individuals with type 2 diabetes have an altered metabolic profile

Table 1 summarises the cardiometabolic risk factors in the study population. Sex proportions in each of the study groups were not significantly different, although there were 63% of women in the healthy group and 41% in the type 2 diabetes group. Participants with type 2 diabetes were older than healthy participants (54 ± 7 vs 47 ± 10 years) and had higher BMI (*p*<0.01), with a median of 23.6 kg/m^2^ in the healthy participants and 28.4 kg/m^2^ in the group with type 2 diabetes. Therefore, the statistical analysis of all parameters was corrected for age and BMI. The type 2 diabetes group displayed the classic alterations associated with diabetes in their metabolic profile, namely elevated levels of fasting glucose (FG; *p*<0.05), HbA_1c_ (*p*<0.001) and HOMA-IR (*p*<0.05) and reduced HDL-cholesterol (*p*<0.01) compared with the healthy group. However, total cholesterol (*p*<0.05) and LDL-cholesterol (*p*<0.001) were lower in participants with type 2 diabetes compared with healthy participants, possibly due to the statin treatments that approximately 70% of the type 2 diabetic participants were taking (see ESM Table 3). High-sensitive C-reactive protein (hsCRP) levels and leukocyte and neutrophil counts were higher in the type 2 diabetic participants (all *p*<0.05), reflecting the inflammatory state associated with the disease.

**Table 1:** Cardiometabolic risk factors and inflammatory parameters in the study population

Parameter	Healthy participants	Type 2 diabetes group	*p* value	Age- and BMI-corrected *p* value
*n*	28	25	–	–
Sex (% women)	63	41	NS	–
Age (years)	47 ± 10	54 ± 7	<0.001	–
BMI (kg/m^2^)	23.6 (20.5–26.1)	28.4 (26.4–30.5)	<0.01	–
FG (mmol/l)	4.88 (4.55–5.27)	6.66 (5.22–7.88)	<0.001	<0.05
HbA_1c_ (mmol/mol)	34 (32–38)	46 (40–58)	<0.001	<0.001
HbA_1c_-DCCT (%)	5.28 (5.07–5.59)	6.36 (5.78–7.42)	<0.001	<0.001
Insulin (pmol/l)	39.59 (30.56–68.76)	74.31 (49.31–100.01)	<0.01	NS
HOMA-IR	1.22 (0.86–2.24)	2.69 (2.10–4.96)	<0.001	<0.05
Total cholesterol (mmol/l)	4.82 (4.66–5.80)	3.86 (3.32–5.13)	<0.001	<0.05
HDL-c (mmol/l)	1.53 (1.17–1.84)	1.19 (1.04–1.50)	<0.01	<0.01
LDL-c (mmol/l)	3.06 (2.56–3.86)	1.86 (1.29–2.62)	<0.001	<0.001
Triacylglycerols (mmol/l)	0.89 (0.62–1.50)	1.13 (0.79–2.17)	<0.05	NS
hsCRP (mg/l)	0.73 (0.40–2.88)	1.85 (0.72–4.39)	<0.05	<0.05
Leukocyte count (×10^9^/l)	6.4 (5.3–6.9)	7.4 (6.1–8.9)	<0.01	<0.05
Neutrophil count (×10^9^/l)	3.3 (2.8–4.3)	4.3 (3.6–5.8)	<0.01	<0.05
Lymphocyte count (×10^9^/l)	1.9 (1.6–2.4)	2.1 (1.7–2.5)	NS	NS
Monocyte count (×10^9^/l)	0.5 (0.4–0.7)	0.5 (0.4–0.6)	NS	NS
Eosinophil count (×10^9^/l)	0.2 (0.1–0.2)	0.2 (0.1–0.2)	NS	NS

Normally distributed parameters are shown as mean ± SD, while non-normally distributed data are expressed as medians (IQR). Comparisons between groups were performed using an independent-samples *t* test or Mann–Whitney *U* test for normally and non-normally distributed data, respectively. The χ^2^ test was used to compare proportions. The influence of age and BMI was tested and corrected using a general linear model

HDL-c/LDL-c, HDL-cholesterol/LDL-cholesterol; NS, not significant

### Circadian rhythm markers are altered in leukocytes from individuals with type 2 diabetes

**Fig. 1:**
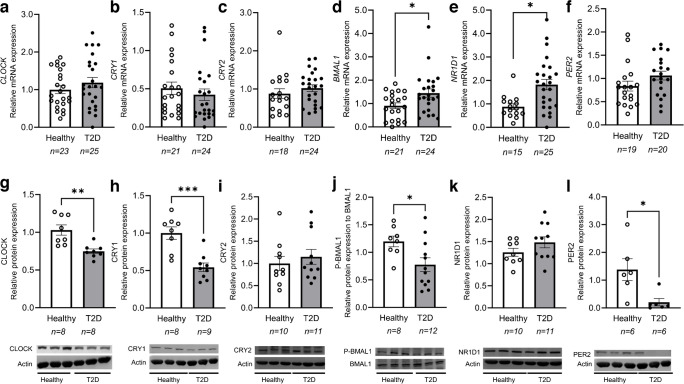
Circadian clock alterations in leukocytes from participants with type 2 diabetes (T2D) compared with healthy participants. (**a**–**f**) Relative mRNA expression in leukocytes from healthy participants and T2D participants: mRNA levels were normalised to 18S expression and to the healthy group levels: (**a**) *CLOCK*, (**b**) *CRY1*, (**c**) *CRY2*, (**d**) *BMAL1*, (**e**) *NR1D1*, (**f**) *PER2*. (**g**–**l**) Protein expression analysis by western blot for (**g**) CLOCK, (**h**) CRY1, (**i**) CRY2, (**j**) p-BMAL1, (**k**) NR1D1 and (**l**) PER2. Data were normalised to actin expression (or to BMAL1 for p-BMAL1) and to the healthy group protein levels. The same controls were used to normalise the protein signal in (**h**) and (**l**) and in (**i**) and (**k**), due to the limited amount of sample (see details in Methods section). Age-adjusted *p* values are shown: *p<0.05, **p<0.01 and ***p<0.001

The subgroup of participants whose leukocytes were used for protein analyses displayed similar characteristics to those of the overall cohort (ESM Table 2). While there were no differences between groups for *CLOCK* and *CRY1* at the mRNA level (Fig. 1a, b), their protein levels were significantly lower in the type 2 diabetes participants than among healthy participants (Fig. 1g, h; *p*<0.01 and *p*<0.001, respectively). We found significantly reduced *CRY1* mRNA levels in older diabetes participants (>50 years old) vs healthy participants in the same age group (ESM Fig. 1, *p*<0.01)*.* There were no significant differences between groups for CRY2 at either the mRNA or protein levels (Fig. 1c, i). *BMAL1* gene expression levels were higher in leukocytes from the participants with type 2 diabetes group than in those from the healthy participants (Fig. 1d; *p*<0.05). However, when analysed separately in older (>50 years old) and younger (<50 years old) participants, the difference was only significant in the younger subgroup (ESM Fig. 1, *p*<0.05). In contrast, protein levels of the phosphorylated active form of BMAL1 (p-BMAL1) were lower in the type 2 diabetes group (Fig. 1j; *p*<0.05). mRNA levels for *NR1D1* were higher in the type 2 diabetes group (Fig. 1e; *p*<0.05), which was due to differences among the older participants but not in the younger subgroup (ESM Fig. 1), but NR1D1 protein levels were unchanged vs healthy participants (Fig. 1k). *PER2* mRNA levels were unaltered in the type 2 diabetes group (Fig. 1f), but a significant decrease in PER2 protein expression was observed in the type 2 diabetes group (Fig. 1l; *p*<0.05).

### mRNA/protein levels for circadian rhythm markers are associated with changes in metabolic profile

**Fig. 2:**
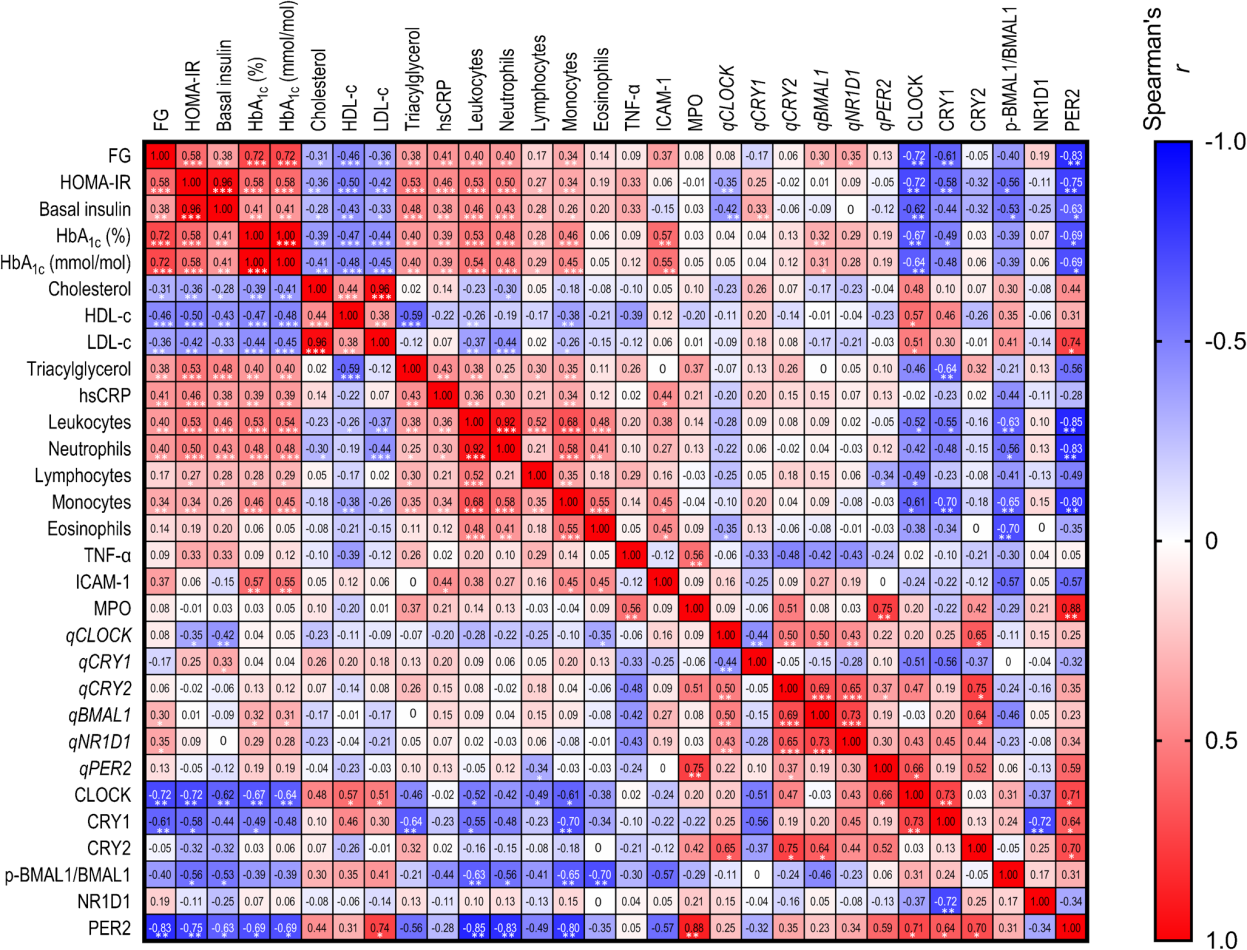
Correlation of clinical parameters and inflammatory markers and gene/protein expression levels for diabetic and healthy participants (total *n*=53). Numbers show the exact Spearman *r* result, while colours represent negative correlations (blue) and positive correlations (red). Asterisks indicate statistically significant differences: *p<0.05, **p<0.01 and ***p<0.001. HDL-c/LDL-c, HDL-cholesterol/LDL-cholesterol; the ‘q’ prefix corresponds to RT-qPCR data (mRNA levels)

Correlation studies were performed for the clinical parameters and circadian gene/protein levels (Fig. 2). As expected, the clinical parameters related to type 2 diabetes (FG, HOMA-IR, basal insulin, HbA_1c_ and triacylglycerols) correlated positively with inflammation markers such as hsCRP and the neutrophil and monocyte counts (*p*<0.01). In contrast, cholesterol, HDL-cholesterol and LDL-cholesterol showed a negative correlation with most of these metabolic and inflammatory parameters (*p*<0.05). For the circadian genes, *CLOCK* mRNA levels correlated negatively with HOMA-IR (*r*=−0.35; *p*<0.01) and basal insulin (*r*=−0.42; *p*<0.01), *CRY1* mRNA levels correlated positively with basal insulin (*r*=0.33; *p*<0.01). *NR1D1* and *BMAL1* mRNA levels showed positive correlations with the metabolic parameters FG (*r*=0.35 and *r*=0.30, respectively; *p*<0.05), and the latter also correlated positively with Hb1Ac (*r*=0.32; *p*<0.01). A negative correlation was found between *CLOCK* mRNA levels and eosinophils (*r*=−0.35; *p*<0.05). *CLOCK* mRNA levels positively correlated with *CRY2*, *BMAL1* and *NR1D1* mRNA levels (*r*=0.50, *r*=0.50 and *r*=0.43, respectively; all *p*<0.01) and negatively with *CRY1* mRNA levels (*r*=−0.44, *p*<0.01), while *CRY2* mRNA levels correlated positively with mRNA levels for *BMAL1* (*r*=0.69; *p*<0.001), *NR1D1* (*r*=0.65; *p*<0.001) and *PER2* (*r*=0.37; *p*<0.05). Finally, *NR1D1* mRNA levels positively correlated with *BMAL1* mRNA levels (*r*=0.73, *p*<0.001). Some of the clinical parameters displayed a negative correlation with the circadian rhythm proteins CLOCK, CRY1, p-BMAL1 and PER2. In particular, CLOCK, CRY1 and PER2 correlated negatively with FG (*r*=−0.72, *p*<0.01; *r*=−0.61 and *r*=−0.83, *p*<0.05, respectively) and HOMA-IR (*r*=−0.72, *p*<0.01; *r*=−0.58, *p*<0.05 and *r*=−0.75, *p*<0.01; respectively). Furthermore, CLOCK and PER2 correlated negatively with basal insulin (*r*=−0.62, *p*<0.01 and *r*=−0.63; *p*<0.05) and HbA_1c_ (*r*=−0.67, *p*<0.01 and *r*=−0.69; *p*<0.05). In contrast, p-BMAL1 correlated negatively with HOMA-IR and basal insulin (*r*=−0.56 and *r*=−0.53, both *p*<0.05).

The total numbers of leukocytes and monocytes correlated negatively with CLOCK, CRY1, p-BMAL1 and PER2 (*p*<0.05). Neutrophil numbers correlated negatively with the circadian proteins p-BMAL1 (*r*=−0.56, *p*<0.05) and PER2 (*r*=−0.83, *p*<0.01), whereas lymphocytes correlated negatively only with CLOCK (*r*=−0.49; *p*<0.05) and eosinophils only with p-BMAL (*r*=−0.70; *p*<0.01). When analysing circadian mRNA–protein correlations, the CRY1 protein correlated positively with CLOCK (*r*=0.73, *p*<0.01) and PER2 (*r*=0.64; *p*<0.05) and negatively with NR1D1 (*r*=−0.72, *p*<0.01). CLOCK also correlated positively with *PER2* mRNA and protein levels (*r*=0.66 and *r*=0.71, respectively; *p*<0.05). A negative correlation was observed between triacylglycerols and CRY1 (*r*=−0.64; *p*<0.01) and positive correlations were observed between CLOCK and HDL-cholesterol (*r*=0.57; *p*<0.01) and LDL-cholesterol (*r*=0.51; *p*<0.05). However, given the lipid-lowering effect of the statin treatments received by some type 2 diabetes participants, these findings for lipid parameters may not reflect the underlying pathophysiology of the disease.

### Type 2 diabetes impairs leukocyte–endothelial interactions

**Fig. 3:**
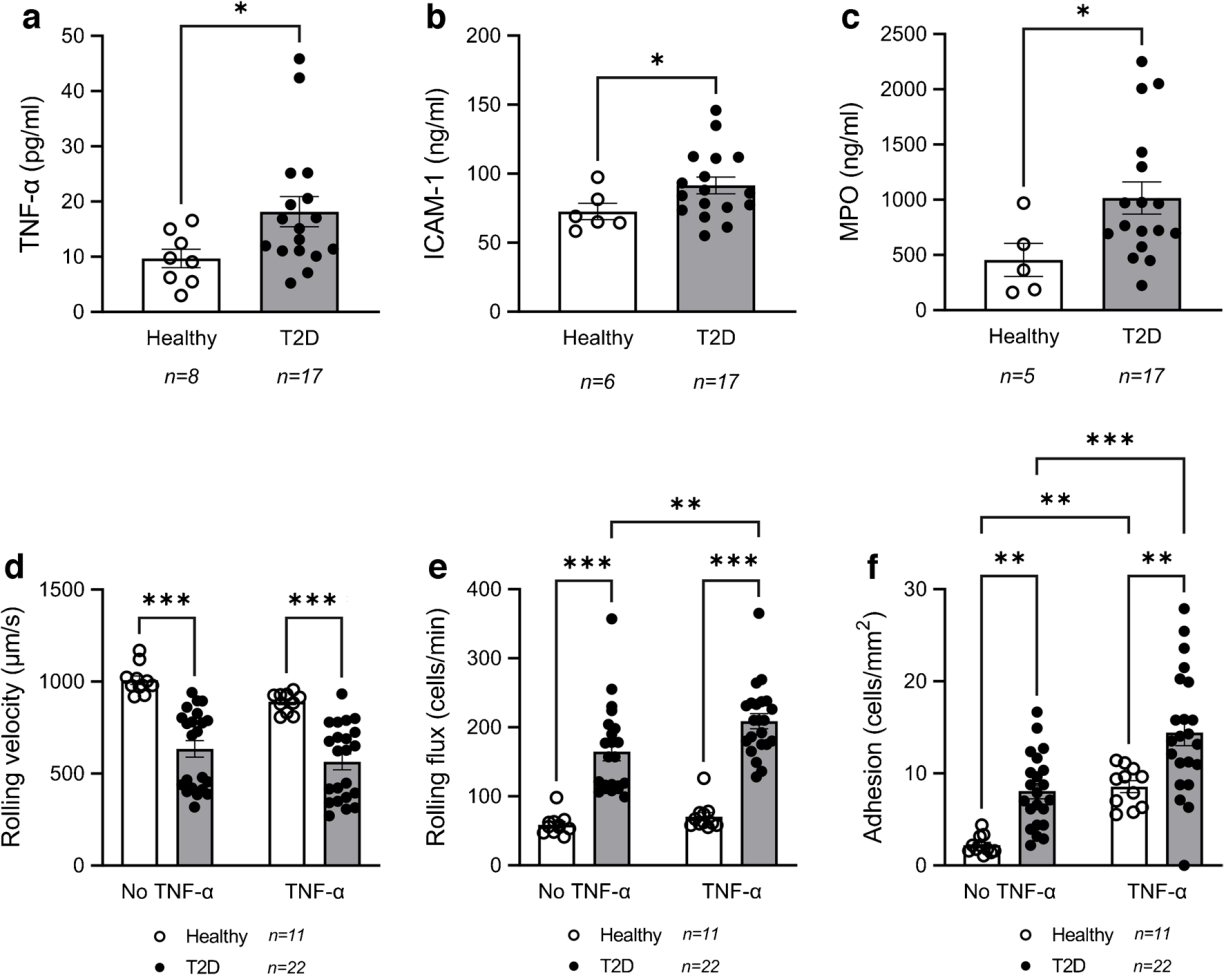
Inflammatory marker levels and leukocyte–endothelium interactions in the absence or presence of TNF-α in participants with type 2 diabetes (T2D) and healthy participants. (**a**–**c**) Inflammatory marker levels: (**a**) TNF-α, (**b**) ICAM-1, (**c**) MPO. (**d**–**f**) Leukocyte–endothelium interactions: (**d**) rolling velocity, (**e**) rolling flux, (**f**) adhesion. Asterisks indicate statistically significant differences: *p<0.05, **p<0.01, ***p<0.001

In order to explore in more depth the inflammatory process that occurs in type 2 diabetes, we measured the levels of inflammation markers TNF-α, ICAM-1 and MPO in serum, and compared the leukocyte–endothelium interactions in type 2 diabetic participants with those in healthy participants. The levels of all three inflammation markers were increased in the diabetes group compared with healthy participants (Fig. 3a–c). In addition, levels of the adhesion molecule ICAM-1 positively correlated with HbA_1c_ (*r*=0.57; *p*<0.01) and hsCRP (*r*=0.44; *p*<0.05) (Fig. 2). MPO levels showed a positive correlation with *PER2* (*r*=0.75; *p*<0.01) (Fig. 2). For leukocyte–endothelial interactions, leukocyte rolling velocity was lower in participants with type 2 diabetes than in healthy participants, while rolling flux and adhesion were higher, showing a higher level of interaction with endothelial cells in type 2 diabetic participants (Fig. 3d–f, *p*<0.01). Interestingly, these interactions intensified when the endothelial cells were treated with TNF-α. In particular, this treatment further increased rolling flux in the type 2 diabetic participants (Fig. 3e;* p*<0.01) and increased adhesion in both healthy and type 2 diabetic participants (Fig. 3e, f; *p*<0.01 for healthy and *p*<0.001, for type 2 diabetes).

### The circadian rhythm inhibitor CLK8 increases leukocyte–endothelium interactions

**Fig. 4:**
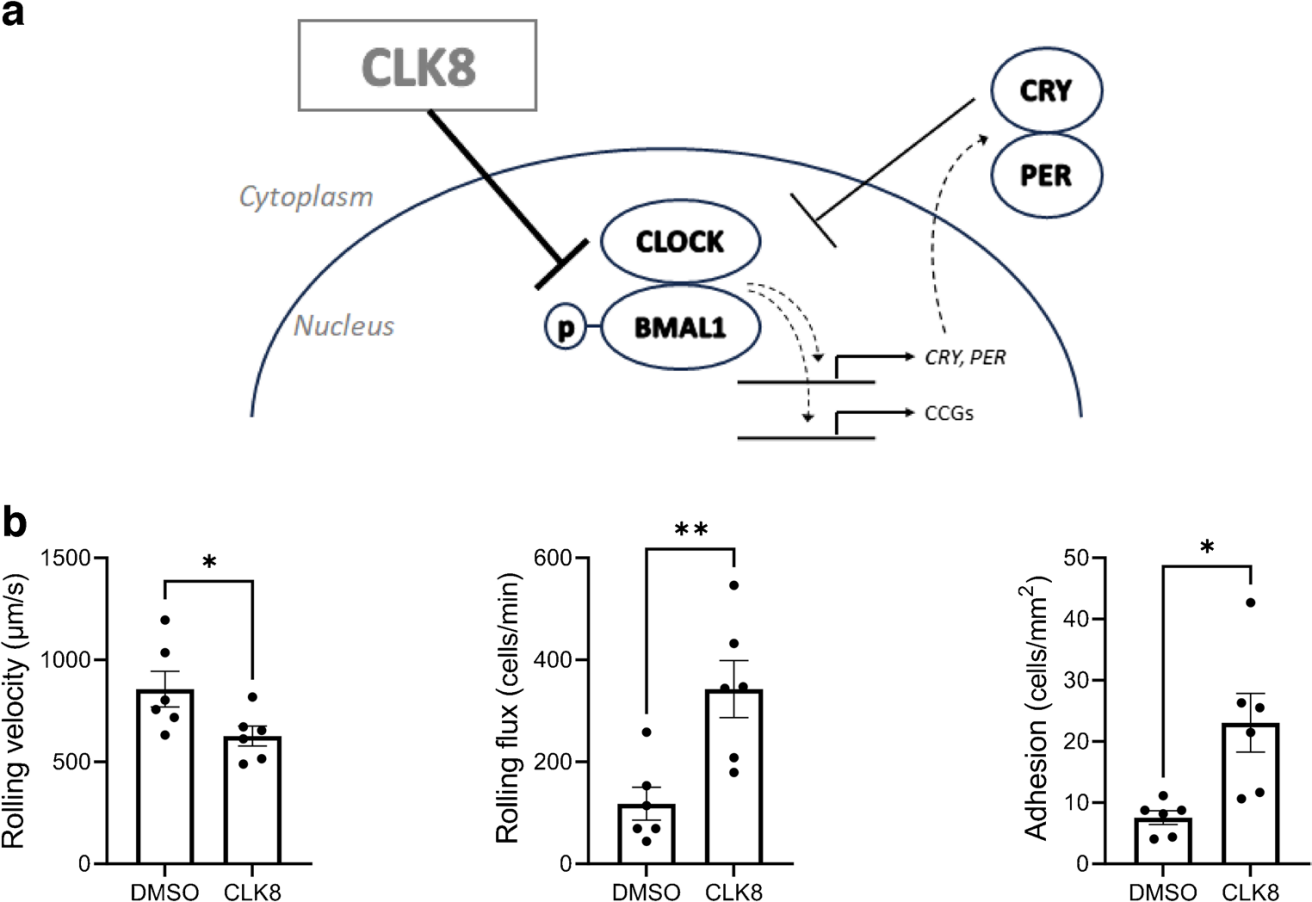
Effects of the CLOCK inhibitor CLK8. (**a**) Mechanism of action of CLK8 on the circadian rhythm modulators. CLOCK/BMAL1 promote *CRY* and *PER* transcription, creating a negative loop, as CRY and PER inhibit CLOCK/BMAL1 activity. CLK8 inhibits the interaction between CLOCK and p-BMAL1, thus reducing the transcription of CCGs (clock-controlled genes) and *CRY* and *PER*, ultimately disrupting the normal negative feedback of the pathway. (**b**) Leukocyte–endothelium interactions in THP-1 cells treated with or without 20 µmol/l CLK8 for 48 h (*n*=6). Asterisks indicate statistically significant differences: *p<0.05, **p<0.01

THP-1 cells (a human monocyte cell line) and the CLOCK/BMAL binding inhibitor CLK8 were used to assess whether impairment of the molecular clock system in leukocytes has an influence on their interaction with endothelial cells. Treatment of THP-1 cells with CLK8 decreased rolling velocity (*p*<0.05) and increased rolling flux (*p*<0.01) and adhesion (*p*<0.05) compared with vehicle conditions (Fig. 4). These results suggest that inhibition of CLOCK/BMAL1 results in an increase in the level of interaction between THP-1 cells and the HUVEC monolayer, similar to the enhanced interactions shown for cells from our participants with type 2 diabetes.

## Discussion

Our data demonstrate a dysregulation of the molecular clock system in PBMCs from participants with type 2 diabetes, manifested by a decrease in CLOCK, CRY1, p-BMAL1 and PER2 protein levels and an increase in *BMAL1* and *NR1D1* mRNA levels. Our type 2 diabetes cohort displayed classic alterations in metabolic profile, including elevated FG, HbA_1c_ and HOMA-IR levels and dyslipidaemia compared with healthy participants. Markers of inflammation, such as counts of various leukocyte types, were elevated in participants with type 2 diabetes. Interestingly, we found several negative correlations between the increases in these parameters and circadian clock protein levels, suggesting crosstalk between circadian clock molecular alterations and metabolic impairment and chronic inflammation. In addition, our type 2 diabetes participants displayed altered subclinical atherosclerotic markers (leukocyte rolling velocity, flux and adhesion), with a pro-atherosclerotic pattern. Alterations in leukocyte–endothelial interactions were mimicked by using a CLOCK/BMAL1 inhibitor (CLK8), which allowed us to demonstrate that circadian clock alterations in immune cells promote enhanced interaction of these cells with the endothelium, a mechanism that may underlie the high atherosclerotic risk associated with type 2 diabetes.

Altered daily rhythms are related to the development of type 2 diabetes and affect heart health, thereby increasing the chances of developing a CVD such as CHD [24–26]. In addition to the central clock, peripheral clocks play important roles in regulating circadian rhythms in various metabolic tissues, e.g. gut, muscle or pancreas, where they regulate whole-body glucose homeostasis [27–29]. Rhythmicity in gene expression has also been described in PBMCs [15].

Previous research suggests that chronic activation of the immune system and a high leukocyte count play a role in type 2 diabetes [30–32]. Increased leukocyte numbers are correlated with the incidence of cardiovascular events by enhancing acute thrombosis and atherosclerosis [33]. Interestingly, the numbers of leukocytes in blood peak during the night, a phenomenon that is driven by circadian control of leukocyte trafficking [34]. Local leukocyte migration is regulated by sympathetic nerves and mediated by rhythmic expression of endothelial cell adhesion molecules and chemokines [35]. Furthermore, the levels of circulating proinflammatory cytokines peak during nocturnal sleep, whereas anti-inflammatory cytokines are preferentially secreted during the daytime [36]. In addition, cytokine secretion from activated macrophages follows circadian oscillations [34]. Circadian variation in susceptibility to myocardial infarction or ischaemic stroke has been described, with a peak in the morning [37, 38]. Therefore, one may speculate that immune cell numbers and inflammatory signalling must be properly regulated in a circadian manner for cardiovascular health to be preserved. Our findings indicate that the molecular clock system in immune cells is altered in participants with type 2 diabetes, and that these alterations occur mainly at the protein level. Previous studies have shown that leukocyte mRNA levels for core clock genes are downregulated in diabetic vs control participants [39, 40]. Importantly, ageing is a relevant factor when studying circadian gene expression levels. Indeed, in a study by Ando et al [39], when individuals with diabetes were compared with younger non-diabetic individuals, the expression levels of *CLOCK*, *BMAL1*, *PER2*, *PER3* and *CRY1* did not differ in the morning (at 09:00 hours), and only *PER1* was significantly decreased in those with diabetes. In contrast, when an age-matched non-diabetes group was used for comparisons, diabetes patients displayed significant reductions in *BMAL1*, *PER1* and *PER3*. As our study did not include an age-matched control group, we subdivided participants both with and without diabetes into a younger group (<50 years) and an older group (>50 years) to analyse mRNA levels. *BMAL1* mRNA levels increased in the younger diabetes participants but not in the older ones compared with the corresponding healthy participants. In contrast, *NR1D1* mRNA levels increased specifically in the older diabetic group, who also exhibited significantly reduced *CRY1* mRNA levels. A study by Yu et al [40] also reported decreased mRNA levels for clock genes in the peripheral blood cells of individuals with diabetes, which correlated with poorer glycaemic control and increased levels of proinflammatory cytokines. However, none of these studies measured circadian protein levels: to our knowledge, our study is the first to assess post-transcriptional changes in the leukocyte molecular clock system. Regulation of clock protein levels would control the cell’s circadian phase more effectively than transcript levels, as non-transcriptional mechanisms are sufficient to sustain circadian timekeeping in eukaryotes [41]. Levels of the core clock proteins assessed in our study – CRY1, CLOCK, PER2 and p-BMAL1 (the active form of BMAL1) – were significantly lower in type 2 diabetic participants than in healthy participants. This reduction was independent of age, as statistical differences remained after adjusting for this confounding factor. However, the transcript levels of these genes were unchanged in the type 2 diabetes group vs healthy participants, except for *BMAL1*, whose levels were higher in the former group. Differences between the mRNA levels and protein levels of these circadian genes may suggest post-transcriptional or post-translational regulation. Upregulated *BMAL1* expression may be influenced by a secondary loop involving receptor-related orphan receptor (ROR) and NR1D1, whose expression is controlled by CLOCK/BMAL1 and which themselves regulate *BMAL1* expression levels [42]. However, we did not observe significant changes in the protein levels of NR1D1. Several factors may have influenced our results. First, protein synthesis is altered under many conditions and by ageing [43], and the reduced translation of circadian gene peaks has been proposed as one of the mechanisms underlying the loss of circadian rhythm in ageing. Second, glucose has been proven to alter mRNA translation [44]. However, the differences found in the molecular clock between our diabetic participants and healthy participants were maintained after adjustments for age and BMI.

Intriguingly, we found several associations between downregulated clock protein levels and the altered metabolic and inflammatory profile in type 2 diabetes participants, in accordance with the results obtained by Yu et al [40], which suggests that the molecular clock is a key player in the pathophysiology of diabetes and its comorbidities. In particular, downregulation of CLOCK, p-BMAL and CRY1 protein levels is associated with poorer glucose control and increased inflammatory parameters. Given that these are only associations, we sought to clarify the direct mechanism by which alterations in the leukocyte intrinsic clock system lead to a process of increased inflammation that may ultimately precipitate the onset of CVD during diabetes. Specifically, in the present study, we established causality by inhibiting the action of the CLOCK/BMAL1 complex, having observed that leukocyte–endothelial interactions were enhanced in a similar way to that seen in type 2 diabetes participants. Future studies should investigate whether genes that regulate leukocyte capture, rolling and extravasation are clock-controlled genes under the control of the CLOCK/BMAL1 complex. Increasing our knowledge about the molecular mechanisms underlying these processes may lead to the discovery of potential targets, applications and therapeutic strategies to prevent or delay the onset of CVD in susceptible populations such as type 2 diabetes patients.

The present study has some limitations, such as the limited number of leukocytes, which meant it was not always possible to perform all the experiments for every participant. In the case of the mRNA/protein studies, mRNA was obtained for all participants, but it was not always possible to obtain proteins. However, all the participants included in the protein studies were also included in the mRNA analysis. Another limitation is that a cell-specific protein/mRNA analysis was not performed. The lack of variation in some of the circadian mRNA expression analyses between type 2 diabetic participants and healthy participants may have been a consequence of these factors.

In summary, the present study is relevant in that we demonstrate a decrease in core clock proteins in the leukocytes of participants with type 2 diabetes and overweight, and a correlation of this decrease with the impaired metabolic and proinflammatory profile seen in type 2 diabetes. CLOCK/BMAL1 inhibition increases leukocyte–endothelial interactions in THP-1 cells, suggesting a role for this machinery in acceleration of the inflammatory process. Altogether, our results help to understand the underlying mechanisms of onset of CVD in type 2 diabetes, and thus represent a step towards the development of new targets and therapeutic strategies to address this pathology.

## Supplementary Information

Below is the link to the electronic supplementary material.

Supplementary file1 (PDF 1.15 MB)

## Data Availability

The datasets used and/or analysed during the current study are available from the corresponding authors on reasonable request.

## Abbreviations

BMAL1: Basic helix-loop-helix ARNT like 1
CLOCK: Circadian locomotor output cycles kaput
CRY1/2: Cryptochrome 1/2
FG: Fasting glucose
hsCRP: High-sensitive C-reactive protein
ICAM-1: Intercellular adhesion molecule 1
MPO: Myeloperoxidase
NR1D1: Nuclear receptor subfamily 1 group D member 1
PBMCs: Peripheral blood mononuclear cells
PER: Period circadian protein homologue
