# Supplementary material for: Molecular circadian clock disruption in the leukocytes of individuals with type 2 diabetes and overweight, and its relationship with leukocyte–endothelial interactions
Source: Diabetologia. 2024 Jul 10;67(10):2316–28. doi: 10.1007/s00125-024-06219-z (PMC11446997; doi:10.1007/s00125-024-06219-z)
Supplement: Supplementary file 1 — Supplementary file1 (PDF 1.15 MB) [file 125_2024_6219_MOESM1_ESM.pdf]

ESM Table 1. Sequences of primers used for qPCR

**Gene**	**Sequence accession number**	**Sequence (5'→3')**	**Amplicon length (bp)**
BMAL1	NM_001178	F: GCTCAGGAGAACCCAGGTTATC<br>R: GCATCTGCTTCCAAGAGGCTCA	161
CLOCK	NM_004898	F: CAGGCAGCATTTACCAGCTCATG<br>R: GTAGCTTGAGACATCACTGGCTG	120
CRY1	NM_004075	F: GCAGTTGCTTGCTTCCTGACAC<br>R: GACAGCCACATCCAACTTCCAG	125
CRY2	NM_021117	F: AGGAGAACCACGACGAGACCTA<br>R: CCGTTCCAAGTGCTTATCCAGG	134
NR1D1	NM_021724.5	F: CTGCCAGCAATGTCGCTTCAAG<br>R: TGGCTGCTCAACTGGTTGTTGG	150
PER2	NM_022817.3	F: GCGTGTTCCACAGTTTCACC<br>R: GCGGATTTCATTCTCGTGGC	146
18S	NR_003286	F: ACCCGTTGAACCCCATTCGTGA<br>R: GCCTCACTAAACCATCCAATCGG	159

Sequences of primers for the analysis of the clock genes by RT-qPCR. Primers were designed with NCBI BlastN tool using the accession numbers above and sequences are displayed from the 5' to 3' ends. Amplicon length was set below 200bp. F= forward; R= reverse

ESM Table 2. Characteristics of patients whose leukocytes were used for western blot.

Parameter	Healthy participants	Type 2 diabetes group	p-value	Age and BMI corrected p-value
N	9	12	-	-
Sex (% women)	44.4	16.7	ns	-
Age (years)	4.81 ± 0.94	3.20 ± 0.56	<0.01	-
BMI	24.0 ± 2.6	28.2 ± 1.9	<0.01	-
FG (mmol/l)	86.7 ± 6.8	129 ± 24	<0.001	<0.01
HbA1c (mmol/mol)	34.0 (30.0-36.0)	58.0 (38.5-60.0)	<0.001	<0.05
HbA1c-DCCT (%)	5.24 (4.88-5.44)	7.42 (5.68-7.66)	<0.001	<0.05
Insulin (pmol/l)	48.62 (30.21-65.98)	67.37 (48.96-82.65)	ns	<0.01
HOMA-IR	1.61 (0.90-2.09)	2.28 (2.17-3.80)	<0.05	<0.01
Total cholesterol (mmol/l)	4.95 ± 1.01	3.57 ± 1.01	<0.01	ns
HDL-c (mmol/l)	1.50 ± 0.38	1.12 ± 0.22	<0.01	<0.05
LDL-c (mmol/l)	2.62 (2.37-4.04)	1.68 (1.02-2.14)	<0.001	<0.01
Triacylglycerol (mmol/l)	0.68 (0.58-1.31)	1.07 (0.82-1.95)	ns	ns
hsCRP (mg/l)	0.99 (0.34-2.13)	1.65 (0.55-2.44)	ns	ns
Leukocyte count (*10^9/l)	6.40 (5.10-6.60)	9.2 0 (7.70-10.55)	<0.01	<0.01
Neutrophil count (*10^9/l)	3.00 (2.55-4.00)	5.40 (4.00-6.55)	<0.01	<0.01
Lymphocyte count (*10^9/l)	1.90 (1.75-2.45)	2.50 (1.75-3.10)	ns	ns
Monocyte count (*10^9/l)	0.40 (0.40-0.40)	0.70 (0.50-1.00)	<0.001	<0.01
Eosinophil count (*10^9/l)	0.10 (0.10-0.10)	0.20 (0.20-0.50)	<0.05	ns

Normally distributed parameters are shown as mean ± standard deviation, while non-normally distributed data are expressed as medians (25th–75th quartiles). Comparisons between groups were carried out using the independent samples t-test or Mann Whitney U test for normally and non-normally distributed data, respectively. The χ^2 test was used to compare proportions. The influence of age and body-mass index (BMI) was tested and corrected by a general linear model. ns: not significant. FG: fasting glucose, hsCRP: high-sensitive C-reactive protein.

ESM Table 3. Proportions of patients receiving various treatments

Treatment	Healthy participants	Type 2 diabetes group (%)
Antidiabetic drugs		
Insulin	-	35.90
Insulin + Metformin	-	25.64
Insulin + DPP4 inhibitors	-	15.38
Insulin + SGLT2 inhibitors	-	35.90
Insulin + GLP-1RA	-	20.51
Metformin	-	53.85
Metformin + DPP4 inhibitors	-	12.82
Metformin + SGLT2 inhibitors	-	38.46
Metformin + GLP-1RA	-	30.77
DDP4 inhibitors	-	20.51
SGLT2 inhibitors	-	71.79
GLP-1RA	-	51.28
Glitazones		10.26
Glinides		0
Lipid-lowering medication		
Statins	-	69.23
Ezetimibe	-	0
Statins + Fibrates	-	2.56
Statins + Ezetimibe	-	2.56
Antihypertensive medication	-	30.77

Summary of the treatments of type 2 diabetes patients divided in categories. Results are expressed as percentage (%) of patients with each treatment.

ESM Figure 1. mRNA expression in groups by age.

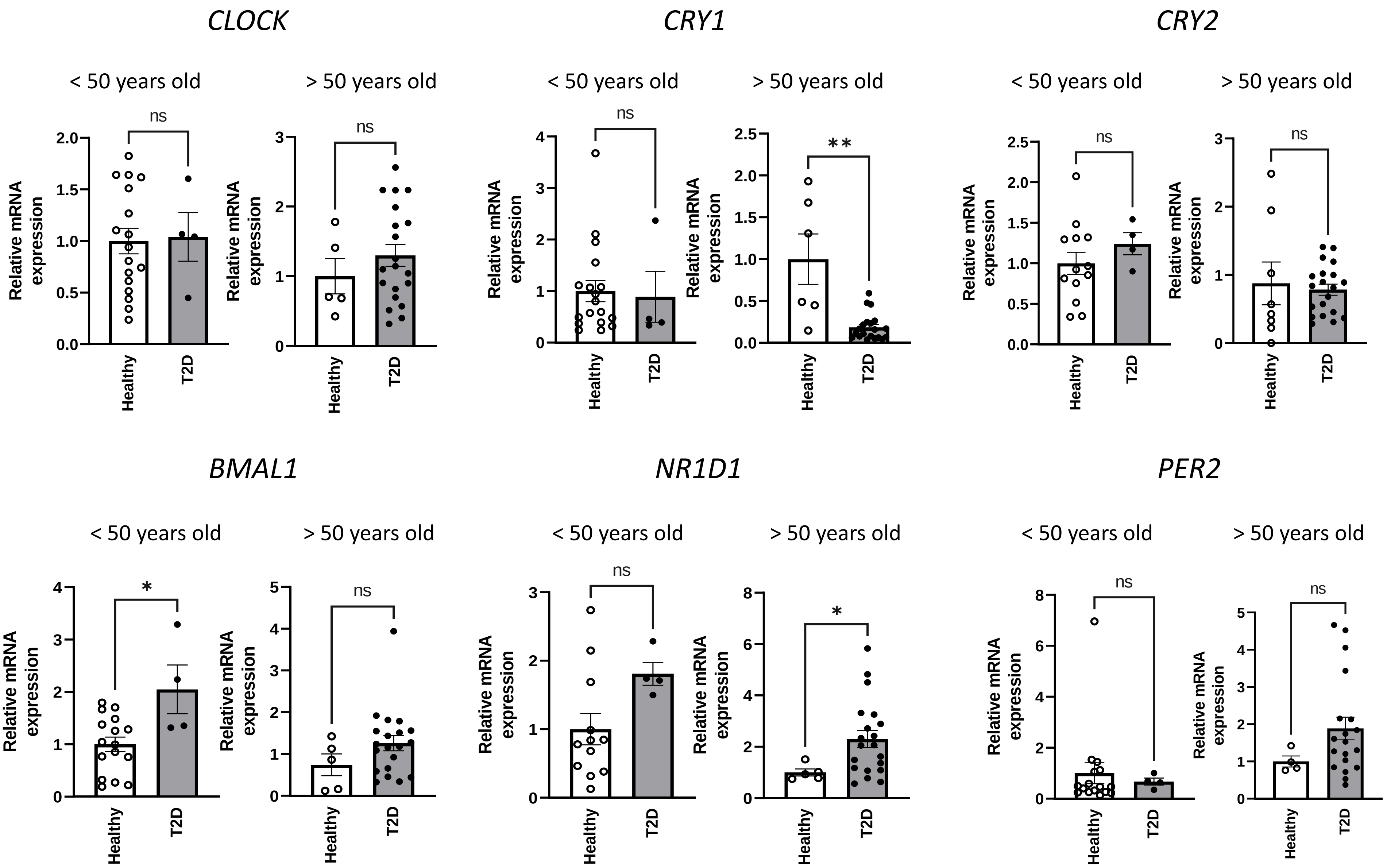

Circadian clock alterations in leukocytes from type 2 diabetes patients compared to healthy subjects. Relative mRNA expression in leukocytes from healthy subjects and type 2 diabetes (T2D) patients divided by age (below and above 50 years old. mRNA levels normalized to 18S expression and to the healthy group levels. *p<0.05, **p<0.01 and ***p<0.001.
